# Structures of human CCL18, CCL3, and CCL4 reveal molecular determinants for quaternary structures and sensitivity to insulin degrading enzyme

**DOI:** 10.1016/j.jmb.2015.01.012

**Published:** 2015-01-28

**Authors:** Wenguang G Liang, Min Ren, Fan Zhao, Wei-Jen Tang

**Affiliations:** 1 Ben-May Department for Cancer Research, The University of Chicago, IL 60637

**Keywords:** chemokine, M16 metalloprotease, crystallography, SAXS

## Abstract

CC chemokine ligands (CCL) are 8-14 kDa signaling proteins involved in diverse immune functions. While CCLs share similar tertiary structures, oligomerization produces highly diverse quaternary structures that protect chemokines from proteolytic degradation and modulate their functions. CCL18 is closely related to CCL3 and CCL4 with respect to both protein sequence and genomic location, yet CCL18 has distinct biochemical and biophysical properties. Here, we report a crystal structure of human CCL18 and its oligomerization states in solution based on crystallographic and small angle X-ray scattering (SAXS) analyses. Our data shows that CCL18 adopts an α-helical conformation at its N-terminus that weakens its dimerization, explaining CCL18’s preference for the monomeric state. Multiple contacts between monomers allow CCL18 to reversibly form a unique open-ended oligomer different from those of CCL3, CCL4, and CCL5. Furthermore, these differences hinge on proline 8, which is conserved in CCL3 and CCL4, but is replaced by lysine in human CCL18. Our structural analyses suggest that a proline 8 to alanine mutation stabilizes a type I β-turn at the N-terminus of CCL4 to prevent dimerization but prevents dimers from making key contacts with each other in CCL3. Thus, the P8A mutation induces depolymerization of CCL3 and CCL4 by distinct mechanisms. Finally, we used structural, biochemical, and functional analyses to unravel why insulin-degrading enzyme (IDE) degrades CCL3 and CCL4 but not CCL18. Our results elucidate the molecular basis for the oligomerization of three closely related CC chemokines and suggest how oligomerization shapes CCL chemokine function.

## Introduction

Protein self assembly underlies numerous biological processes, such as the polymerization of actin and tubulin for intracellular transport and cell motility, or that of amyloidogenic peptides in neurodegenerative diseases [1, 2]. CC chemokine ligands (CCL) comprise 8-14 kDa chemotactic cytokines (chemokines) involved in diverse immune functions [3, 4]. Accumulating data strongly support roles for quaternary structure in regulating CCLs during inflammation and infection [5-9]. Extensive structural analyses reveal that most chemokines share a common core structure: an N-terminal extension followed by a 3_10_ helical turn, an anti-parallel three-stranded β-sheet, and an α-helix [8-10]. CC chemokines also readily dimerize, primarily through the formation of an anti-parallel β-sheet at their N-termini [8, 9]. They can further form higher molecular weight (MW) complexes in the presence of glycosaminoglycans (GAGs), sulfonated polysaccharides present on proteoglycan and extracellular matrices [11]. Certain chemokines, e.g. CCL3, CCL4, and CCL5 form polydisperse, high MW reversible polymers in excess of 600 kDa without the assistance of GAG [12, 13]. The structural bases of how these chemokines form an open-ended, rod-shaped polymer were deciphered recently [7, 14]. Mutational analyses support functional roles for chemokine oligomerization [5-9]. For example, CC chemokine mutants with reduced polymerization fail to increase cell infiltration into the mouse peritoneum [6, 7]. Under flow conditions mimicking blood circulation, depolymerization mutations make CCL3 more effective at arresting monocytes onto activated endothelium [7] but render CCL5 ineffective in CC chemokine receptor, CCR1-mediated cell cycle arrest [5].

Upon secretion, CC chemokines interact with GAGs and proteases during diffusion through the extracellular milieu, transcytosis across the endothelium, and presentation to their targeted receptors on circulating leukocytes. Chemokine dimerization and oligomerization may protect chemokines from proteolytic degradation by extracellular proteases [7-9, 15], since these processes bury chemokine N- and C-termini. However, CCL oligomers are also receptor-binding incompetent because oligomerization buries their receptor-binding sites, particularly their N-termini [8, 16]. Thus, oligomerization prolongs the half-life of these chemokines but reduces the monomer level for the activation of their cognate receptor. Consequently, the presence of equilibrium between monomer, dimer, and high MW oligomers/polymers makes chemokines more effective chemoattractants over a longer range [7]. In addition, the interplay between CC chemokine oligomerization and monomer-specific degradation of CC chemokines has been proposed as a means to encode severity in the chemotactic gradient, thereby guiding a proper chemotactic responses [7]. Additional processes that oligomerization could affect include transcytosis and presentation of CC chemokines, receptor-mediated leukocyte arrest, and chemokine-receptor interaction [8].

CCL18, also known as pulmonary and activation regulated chemokine (PARC) among other names (e.g., MIP-4, DC-CK1, and AMAC-1), is a primate-specific CC chemokine expressed by macrophages, monocytes, and dendritic cells upon infection or inflammation to attract T cells [17-20]. It is constitutively expressed at high levels in lungs and other organs, e.g. placenta and secondary lymphoid organs. It is the most highly expressed chemokine in several human chronic inflammatory diseases, e.g., pneumonitis and pulmonary fibrosis [21, 22] and is a potential biomarker for human pathologies, e.g., Gaucher disease [23]. CCL18 is also highly expressed in tumor-associated macrophages and is thought to be involved in breast cancer metastasis [24]. Despite substantial studies of the biological functions of CCL18, the structure of human CCL18 has not yet been solved.

**Figure 1:**
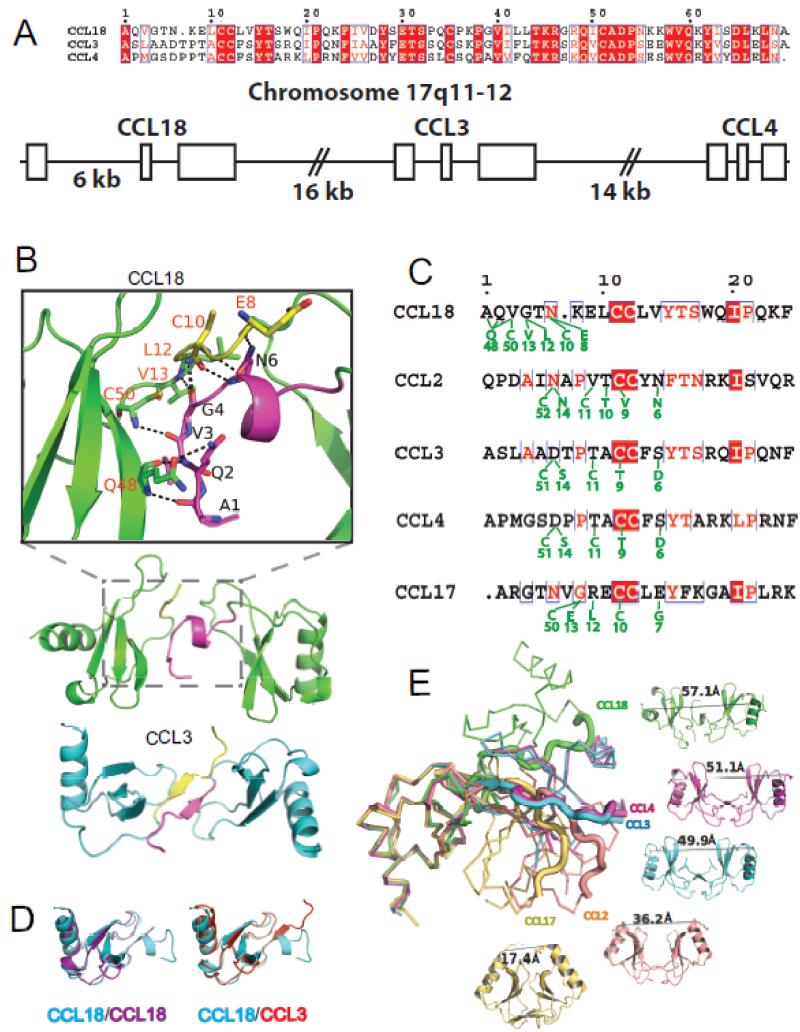
The unique dimer structure of CCL18 A) Sequence alignment and genomic location of CCLs 3, 4, and 18. The three boxes represent the exons of these CCLs and the longer gap between exon 1 and exon2 of CCL18 is highlighted. For simplicity, the gene clusters of these chemokines (LD78α/β/γ for CCL3 and AT744.1/744.2 for CCL4) are not shown. B) Ribbon diagram of CCL18 and CCL3 dimers. Detailed interactions of residues between CCL18 monomers within CCL18 dimer are shown on the top of panel B. The N-termini of CCL18 and CCL3 are highlighted by yellow and magenta. C) Sequence alignment of CCL18, CCL2, CCL3, CCL4 and CCL17. The residues involved in dimerization and hydrogen bonding are depicted in green and conserved sequences are in red. D) Structural alignments between two CCL18 monomers within a CCL18 dimer (left) and between the monomer of CCL3 and CCL18 (right). E) Structural alignment of CCL18, CCL2, CCL3 and CCL17 dimers. PDB codes for CCL18, CCL2, CCL3, CCL4, and CCL17 are 4MHE, 3IFD, 2X69, 2X6L, and 1NR4, respectively.

CCL18 is closely related to macrophage inflammatory proteins, CCL3 (a.k.a. MIP-1α) and CCL4 (a.k.a. MIP-1β), based on primary sequence (Figure 1A). They are 68-69 amino acid-long chemokines that can be secreted by macrophages in response to inflammation and infection. CCL3 and CCL4 are found in most mammals but CCL18 is found only in primates. This suggests a recent evolutionary event from the gene duplication of CCL3 or CCL4, which are located within 40 kb of the human CCL18 gene at chromosome 17q11-12 (Figure 1A) [18, 20] [4, 25, 26]. CCL3 and CCL4 share a common receptor, CCR5, while CCL18 binds to both a conventional G protein-coupled receptor, CCR8 [25] and an atypical receptor, PITPNM3 [24]. CCL3 and CCL4 are quite acidic at physiological pH (pI=4.8). However, like other CC chemokines, CCL18 is quite basic at the neutral pH (pI=9.2). CCL3 and CCL4 are kept at the low levels until induction by inflammation or infection, but, in addition to being inducible by inflammatory and disease conditions, CCL18 is constitutively expressed at high levels in certain tissues. Proline 8 is conserved in CCL3 and CCL4, but not in CCL18. Mutation of Proline 8 to alanine (P8A) profoundly reduces the polymerization of CCL3 and CCL4 [6, 7, 27]. However, the molecular basis of how P8A mutation leads to depolymerization is unknown. As the oligomerization status of CCL18 in solution is unknown, whether position 8 plays a more general role in determining CCL quaternary structure is also unknown.

CC chemokines are subject to degradation by extracellular proteases that selectively cut CC chemokines, particularly at their N and C termini, though certain proteases do cleave CC chemokines outside the N- or C-termini [15]. Such cleavage usually results in their inactivation but can also enhance activity in some cases [15]. Insulin degrading enzyme (IDE) is a M16 zinc-metalloprotease that selectively degrades amyloidogenic peptides such as amyloid β, peptide vital for the progression of Alzheimer’s disease [28, 29]. We have shown that IDE only degrades monomeric CCL3 by stochastically cutting regions outside the CCL3 N and C-termini thereby rendering this chemokine inactive [7]. However, it is unknown whether IDE degrades CCL4 and/or CCL18 and, if so, how. Here, we apply structural and biochemical analyses to address these questions.

## Results

### Structural and SAXS analysis of human CCL18

We used a fluorescence-based thermal shift assay to identify two buffers, citrate pH 5.5 and bicine pH 9.0, that stabilize CCL18 for crystallization [30]. We next found diffraction-quality crystals in the presence of citrate ion via a high-throughput sparse matrix method and solved the structure of CCL18 at 2.1Å resolution in space group P2_1_ (Table 1, Figure 1B). The overall electron density is excellent and the average B factor of atoms in CCL18 is 29 Å^2^. There are two CCL18 dimers in each asymmetric unit (Figure 1B). The overall CCL18 monomer structure consists of an N-terminal segment (aa 1-21) followed by a 3_10_ helical turn (aa 22-24), an anti-parallel three-stranded β-sheet (aa 25-56), and an α-helix (aa 56-69). The two monomers within the CCL18 dimer adopt distinct structures at their N-termini; one has the N-terminal extension (aa 1-6) followed by an α-helix (aa 7-10) and the other has a largely disordered N-terminus (aa 1-7) (Figure 1B). The N-termini of these two monomers (aa 2-5 in one and aa 8-13 in the other) form a network of hydrogen bonds and van der Waal contacts between their N-termini that bury a substantial surface (~549 Å^2^) (Figure 1B-D). The dimer contacts of CCL18 are distinct from other CCL dimers, which form contacts at their N-terminal region via an anti-parallel β-sheet, exemplified by CCL3 (Figure 1B-D). Thus, despite the overall structural similarity of CCL monomers, changes in the amino acid sequence and contacts at N-termini allow CCL dimers to adopt dramatically different shapes, which likely depict the dynamics of CCL dimers that control the dimerization and oligomerization (Figure 1E) [7]. Indeed, we have previously shown the shape of dimers within the CCL3 and CCL4 polymer structure is different from the CCL3 and CCL4 dimers observed by NMR, suggesting the shape flexibility of CCL dimeric structures could play a role in their oligomerization.

**Figure 2:**
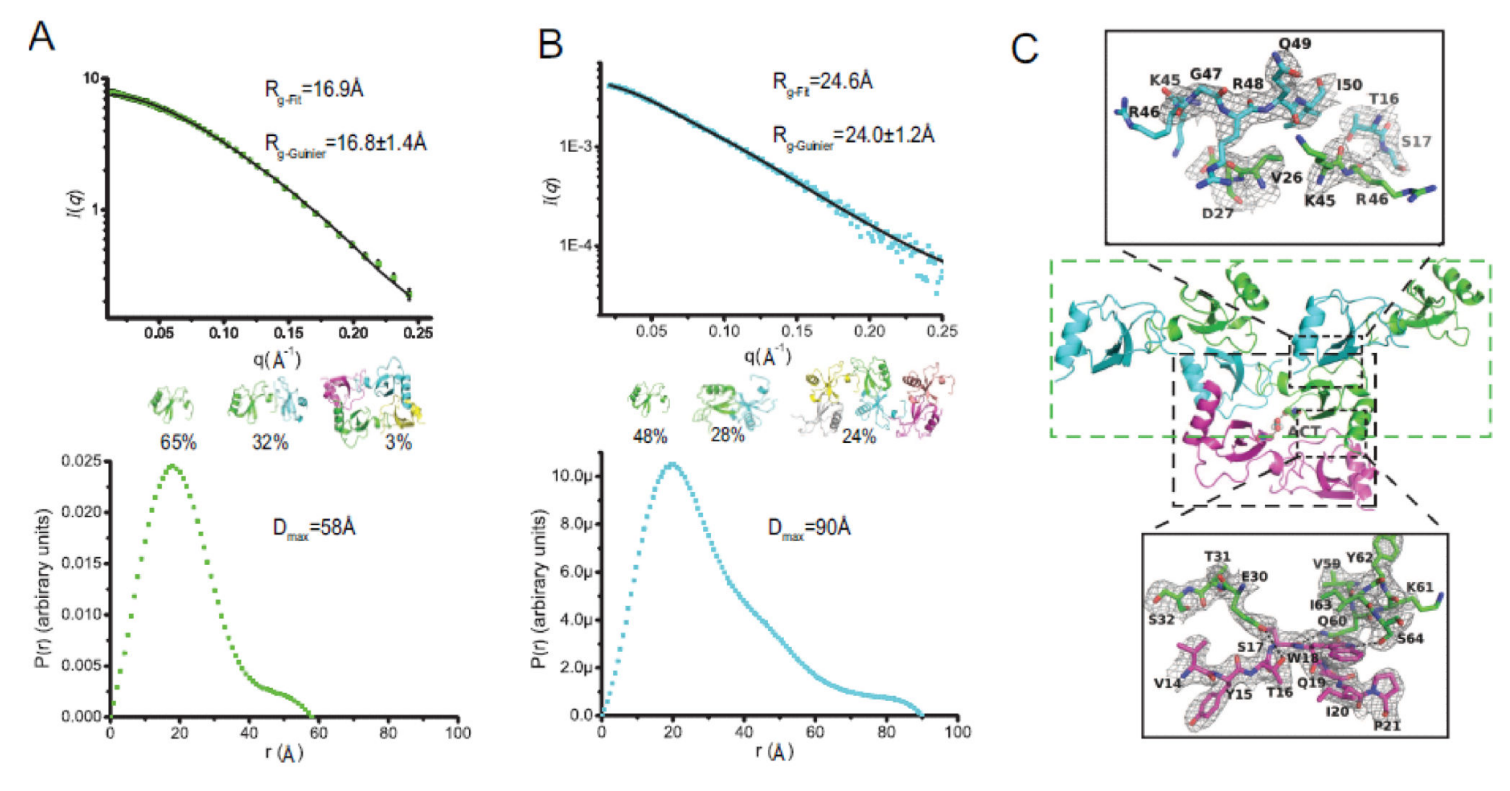
Oligomer structures of CCL18 and SAXS data and analysis CCL18 Scattering curve (top, fitting and R_g_ calculated by program OLIGOMER, experimental R_g_ is calculated by Guinier approximation) and P(r) distribution (bottom, calculated by program GNOM) of CCL18 in (A) 20 mM citrate buffer, pH=5.5 and (B) 20 mM bicine buffer, pH=9.0. 1mg/ml protein was used to collect the SAXS data. Models used to fit the SAXS data were generated from CCL18 structure (4MHE). *χ* was calculated by *Χ*^2^ = [1/(N-1)] Σ_j_{[μI(s_j_)-I_exp_(s_j_)]/σ(s_j_)}^2^ where N is the number of experimental points and μ is the scaling factor. (C) Crystal packaging of CCL18 reveals two distinct oligomer arrangements, tetramer and open-ended polymer. Ribbon representation of possible structures of CCL18 tetramer. The acetate ion (ACT) and coordinating side chains of S32 residues are depicted as CPK. Ribbon representation of possible structures of CCL18 tetramer (C, bottom) and CCL18 hexamer (B, top). Composite iterative-build omit 2mFo-DFc map was calculated with Autobuild in PHENIX and contoured at 1σ. The scattering curve fitting and the fitting R_g_ were performed using Program OLIGOMER. The P(r) distribution and the estimated D_max_ values were calculated using program GNOM.

Since chemokines are known to self-assemble into oligomers, we next addressed the oligomeric state of human CCL18 in solution via SAXS. We first tried SAXS analysis in phosphate buffer saline at pH=7.4. The scattering curve revealed severe aggregation precluding meaningful analysis (Figure S1A). We then chose the citrate buffer at pH=5.5 and bicine buffer at pH=9 for our SAXS studies (Figure 2A-B,S1, Table S1) because, based on our thermal shift assays, these buffers stabilized CCL18 (data not shown). We also chose to analyze SAXS data only at 1 mg/ml CCL18 because our SAXS data showed significant aggregation at a higher concentration (1.6 mg/ml). At either pH, the scattering curves and derived R_g_ values did not fit well with the predicted curves (based on the high χ values) and R_g_ values for a population composed solely of monomers, dimers, or higher order oligomers (Figure S2A, S2C). We then searched extensively using various probable states of CCL18 derived from the packing of the CCL18 crystal structure (Figure 2C). We sought to identify the most parsimonious composition of states that could still have a satisfactory fit to the experimental data, as assessed by the R_g_ and χ values. We found that the scattering curve and P(r) distribution of CCL18 at pH=5.5 best fit with a mixture of monomer (65%) and dimer (32%) (Figure 2A). In order to fit the observed R_g_ (16.8 Å calculated from Guinier approximation) and D_max_ (D_max_=58Å from P(r) distribution), we incorporated 3% CCL18 tetramer found in the asymmetric unit of the CCL18 structure. Such a tetramer should be stable because it has networks of hydrogen bonds and van der Waal contacts with a 982Å^2^ buried surface at the dimer-dimer interfaces (Figure 2C). Alternative tetramers of CCL18 failed to offer a similar fit (Figure S2B). We also tested the tetramer from the structure of CCL3 or CCL5, which have a distinct shape compared to CCL18 (Figure S2B) but they have high R_g_ values for a good fit.

The same strategy was applied to determine the best fit to the scattering profile of CCL18 at pH=9. We found that CCL18 at pH=9 had a larger radius of gyration (24.0 Å calculated from Guinier approximation) and D_max_ (90 Å from P(r) distribution) than CCL18 at pH=5.5 (R_g_=16.7 Å and D_max_=58 Å) (Figure 2B). This indicates that CCL18 at pH=9 forms a larger oligomer than at pH=5.5. Examining the contacts between the symmetry-related CCL18 in our CCL18 structure reveals a 630 Å^2^ buried surface (Figure 2C). This permits the formation of a CCL18 oligomer that can be extended at either end in a zigzag manner (Figure S3). By including this open-ended CCL18 oligomer in our input models, we found that the scattering curve and P(r) distribution of CCL18 at pH=9 produced an optimal fit with a percent composition by conformational state of 48% CCL18 monomers, 28% dimers, and 24% hexamers of the open-ended oligomer (Figure 2B). The incorporation of other probable CCL18 states, including the tetramer observed in the CCL18 population at pH 5.5, or the hexamer from CCL3 or CCL5 failed to generate a good fit to the R_g_ value (Figure S2D). This finding suggests that CCL18 occupies multiple oligomeric states in solution, with the monomeric state being the dominant. Furthermore, these data reveal that the quaternary structure of CCL18 is determined by the pH of its environment. It is worth noting that the arrangement of the open-ended polymer of CCL18 is different from helical shaped polymer structures of the closely related CCL3 and CCL4 [7] and the ladder-shaped structure of CCL5 polymer (Figure S3) [14].

### Structural analysis of the effect of P8A mutation on CCL3 and CCL4

While CCL18 is closely related to CCL3 and CCL4, CCL18 prefers the monomeric state while CCL3 and CCL4 tend to form high MW reversible polymer. One difference among these chemokines is proline 8 residue, which is conserved in CCL3 and CCL4 but occupied by lysine in human CCL18. The change of proline 8 to alanine can profoundly reduce polymerization of CCL3 and CCL4 [7, 27]. The P8A mutation correlates with changes in the ability of CCL3 to recruit leukocytes to the mouse peritoneum and arrest monocytes on the activated endothelium [7]. To address how position 8 controls CCL polymerization, we solved the structure of polymerization-deficient mutants CCL3 and CCL4 P8A at 2.6 Å and 1.6 Å resolution, respectively (Table 1).

**Figure 3:**
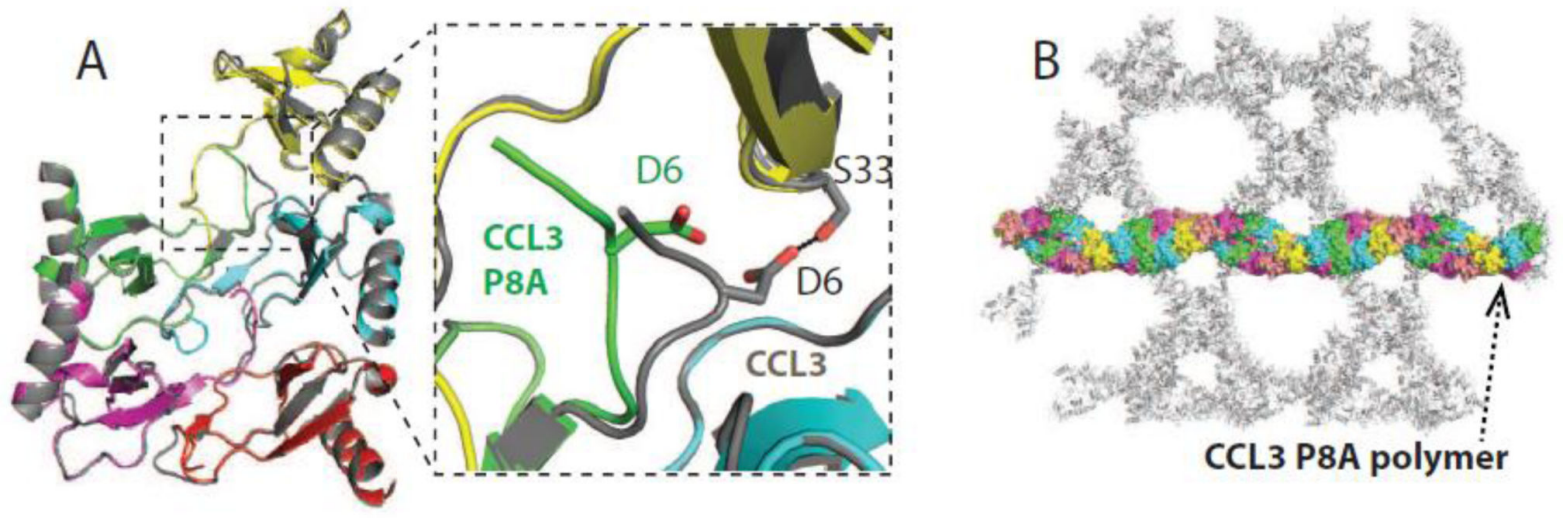
Structures of CCL3 P8A (A) Secondary structure of the CCL3 P8A pentamer (with distinct colors for each monomer) in comparison with CCL3 (gray). The hydrogen bond present in CCL3 but missing in CCL3 P8A is shown in the inset. (B) CCL3 P8A polymer structure shown in the crystal lattice.

CCL3 P8A crystallized in space group P6_2_22. The electron density is in general good except for 1-10 N-terminus loop. The average B factor of atoms in CCL3 P8A is 54 Å^2^. There are five CCL3 molecules in each asymmetric unit (Figure 3A). CCL3 P8A still arranges as a polymer structure similar to wild type CCL3; each dimer rotates 36° to bind to the adjacent dimer such that a decamer completes a 180° turn (Figure 3A, 3B). However, the N-termini of CCL3 P8A exhibit substantial heterogeneity, indicating that these N termini are more disordered than wild type CCL3 and can adopt multiple conformations (Figure 3B). One such conformation results in the loss of a hydrogen bond between D6 and S33 in CCL3 P8A. This disrupts a key contact between CCL3 dimers. The loss of contact between CCL3 dimers and the conformational disorder of CCL3 N-termini likely contribute to the decreased stability of the CCL3 polymer, leading to depolymerization.

**Figure 4:**
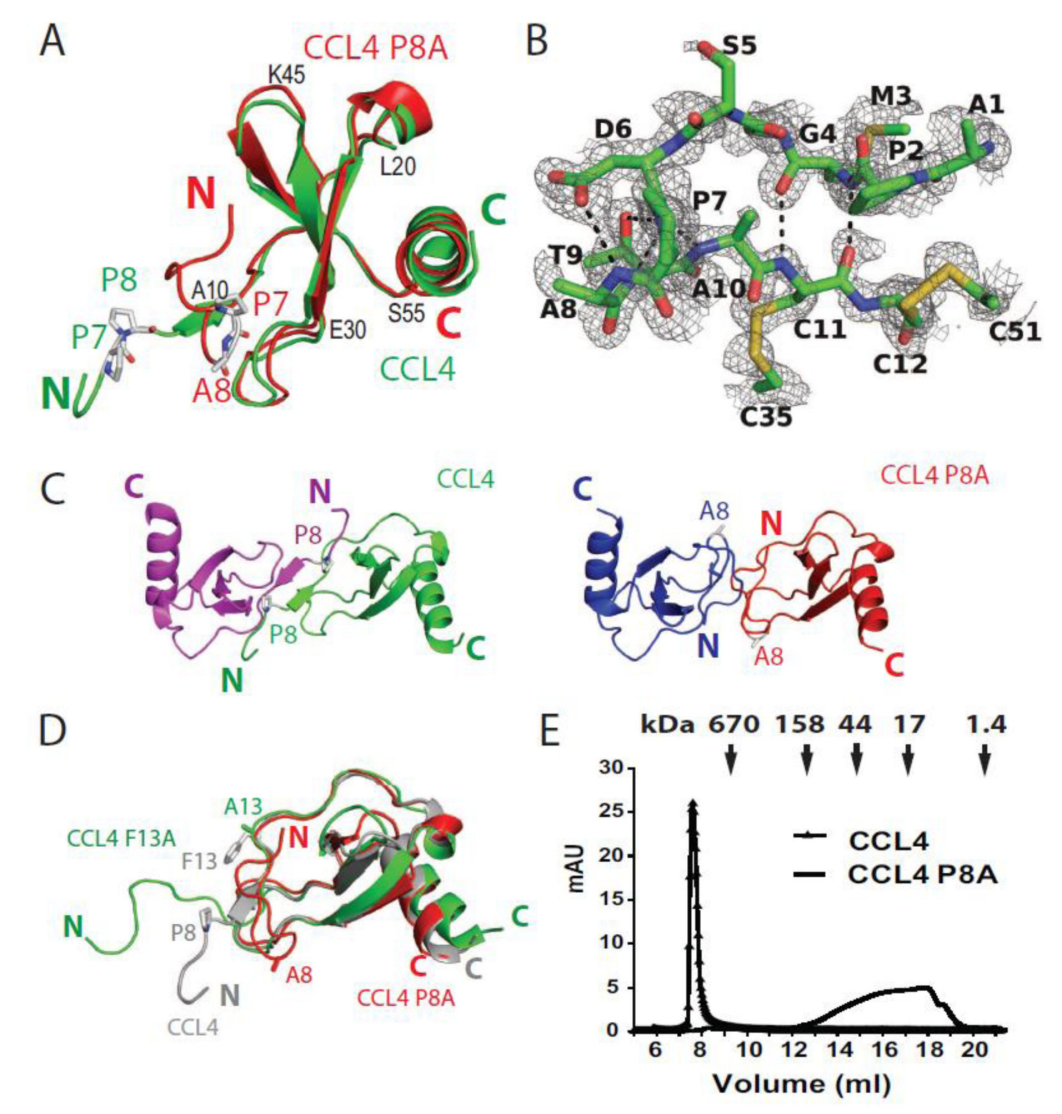
Structure of CCL4 P8A (A) Secondary structure comparison of CCL4 P8A (red) with a CCL4 monomer in the CCL4 polymer structure (green, PDB: 2X6L). Residues 7 and 8 are depicted as sticks. (B) A detailed hydrogen bond network at the N-terminal of CCL4 P8A to prevent its extension and subsequent dimerization. A composite omit 2mFo-DFc map was calculated in PHENIX and contoured at 1σ. (C) Comparison of the CCL4 dimer (top) with the hypothetical model of the CCL4 P8A dimer (bottom) to reveal the steric clash at the N-terminal end of CCL4 P8A in the model of CCL4 P8A dimer. (D) Size exclusion chromatography profile of CCL4 and CCL4 P8A. Chemokines (100 μl 1 mg/ml) were analyzed on Superdex 200 column with buffer containing 20 mM NaCl, 50 mM Tris (pH 7.8) at 4°C. The arrows indicate peak positions of the molecular weight standard. (E) Secondary structure comparison of CCL4 P8A (red, 3TN2), CCL4 F13A (green, 1JE4) and CCL4 (gray; 1HUM). Residues 8 and 13 were depicted as stick. Oxygen, nitrogen, sulfur and carbon atoms are shown in red, blue, yellow and gray, respectively.

The crystal of CCL4 P8A diffracted to 1.6 Å resolution is in space group C2. The electron density is excellent and the average B factor of atoms in CCL4 P8A is 23 Å^2^. Unlike our previously reported crystal structure of the wild type CCL4 polymer [7], the structure of CCL4 P8A is monomeric (Figure 4A). CCL4 P8A maintains the typical chemokine fold (Figure 4A). With respect to residues 9-69, CCL4 P8A is nearly identical to the crystal structure of the CCL4 polymer (2X6L; RMSD = 0.42 Å) [7] and the NMR structure of the CCL4 dimer (1HUM; RMSD = 0.96 Å) [10]. However, CCL4 P8A has a distinct structure at the N-terminus. Instead of the extended loop found in wild type CCL4 [7, 10], the absence of proline at position 8 of CCL4 allows aa 6-9 to form a type 1 β-turn so that the N terminus turns back toward the three-stranded β-sheet of CCL4 (Figure 4A). This permits favorable hydrogen bonds to form between the main chains of residues at the N-terminal loop of CCL4 (Met3 with Cys11; Asp6 with Ala10) (Figure 4B). All prolines (Pro 7 and 8 in wild type and Pro 7 in P8A mutant) are in the trans configuration, thus the formation of type 1 β-turn is not due to the switch of cis-trans peptidyl bonds of proline. The N-terminal conformation of CCL4 P8A would lead to steric clashes between two monomers of CCL4 P8A, which prevents dimerization (Figure 4C), explaining why CCL4 P8A is polymerization deficient. Interestingly, the N-terminus of our CCL4 P8A structure differs from the NMR structure of CCL4 F13A, another CCL4 depolymerization mutant (PDB code=1JE4) (Figure 4D) [27]. CCL4 F13A might resemble the solution structure of CCL4 P8A based on the striking similarity in the representative NMR spectra shown in Kim et al [27]. This suggests that CCL4 P8A may adopt multiple conformations at its N-termini to prevent CCL4 from being oligomerized. Consistent with this hypothesis, our gel filtration data reveals that CCL4 P8A is polydisperse in size and substantially less polymerized in solution compared to wild type CCL4 (Figure 4E).

### The interaction of CCL18 and CCL4 with IDE

We have shown that IDE not only uses the size of the catalytic chamber to selectively degrade CCL3 monomers but also uses the dipolar charge distribution of its catalytic chamber to recognize CCL3 by charge complementarity with its surface [7]. Although CCL18 has high sequence similarity to CCL3, CCL18 has a highly positively charged surface while CCL3 has a highly negatively charged surface, which is consistent with their distinct pI values (9.2 and 4.8, respectively) [7]. We predict that its surface charge distribution will make CCL18 a poor substrate for IDE even though its shape and preference for a monomeric state fit the other IDE substrate requirements. We used MALDI-TOF mass spectrometry to analyze the reaction mixture of CCL18 and IDE in a 200 to 1 ratio after 5-second incubation at 37°C and found no detectable proteolytic fragments of CCL18 (Figure S4). We could not observe any reaction products after a 30-minute incubation (data not shown). However, such prolonged incubation also led to the precipitation of CCL18, which could prevent degradation of CCL18 by IDE.

**Figure 5:**
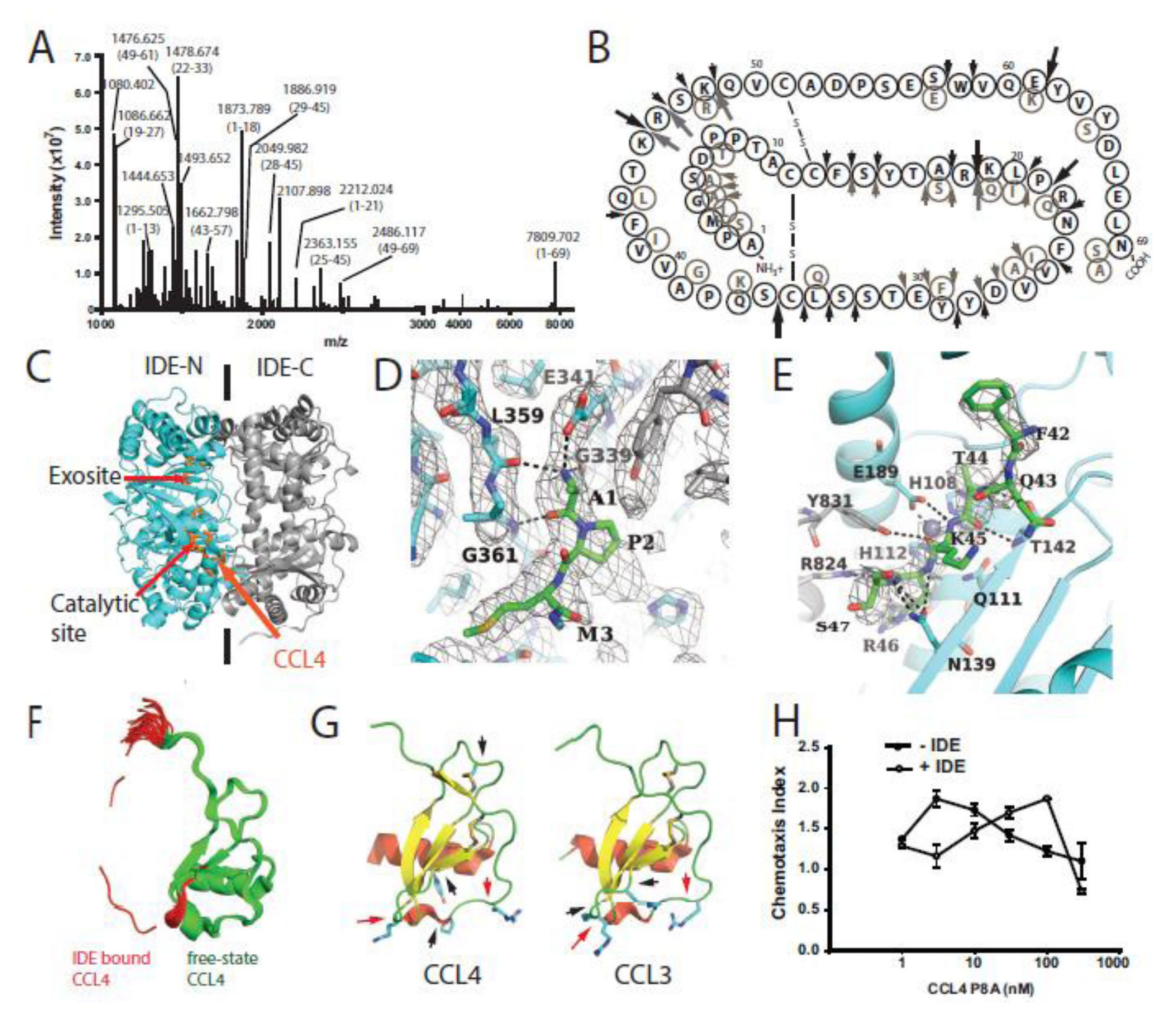
Characterization of the degradation of CCL4 and CCL4 P8A by IDE and functional consequence (A) Representative ESI-FT-ICR-MS spectrum of CCL4 after a 2-min IDE digestion. The identity of the indicated peaks is determined by the mass match of the primary ion (most <5 ppm) and secondary b and y ions (Table S2). (B) Summary of cleavage sites of CCL4 by IDE primary sequence obtained by mass spectrometry analysis is illustrated. Big arrows represent the cleavage sites found in multiple fragments and small arrows indicate those found once (Table S2). Grey and black arrows are the cleavage sites of CCL4 and CCL3 by IDE, respectively. A small fraction of CCL4 is cleaved between residue 18 and 19 prior to IDE cleavage. (C) Overall secondary structure of IDE-CF-E111Q in complex with CCL4. N- and C-terminal domains of IDE are colored cyan and grey, respectively. CCL4 is colored orange. The detailed interactions of CCL4 (shown in stick representation in green) at (D) the exosite and (E) catalytic site. Composite iterative-build omit 2mFo-DFc map was calculated with Autobuild in PHENIX and contoured at 1σ. (F) Comparison of CCL4 in the free form (green, 1HUN) with IDE-bound form (red). For the comparison, the corresponding regions of CCL4 found in the IDE-bound form are colored in red in the free form of CCL4. (G) Structural comparison of major cleavage sites of CCL3 and CCL4. Red and black arrows are the common and unique sites of the most frequent cleavage sites of CCL3 and CCL4 by IDE, respectively. (H) Effect of IDE on the chemotaxis of CCL4 P8A on THP-1 cells. CCL4 P8A was pre-incubated with IDE at a 50:1 molar ratio at 37°C for 50 min. The reaction was stopped and >95% digestion of CCL4 P8A by IDE was confirmed by MALDI-MS.

Using mass spectrometry, we have previously shown that CCL4 is only effectively degraded by IDE in the presence of a depolymerization agent, heparin (from ~0.4 min^−1^ to ~4 s^−1^) [7]. However, the cleavages CCL4 by IDE have not been characterized. We thus identified the cleavage sites of CCL4 by IDE using liquid chromatography-electrospray ionization-Fourier transform ion cyclotron resonance mass spectrometry in conjunction with collision induced dissociation in the gas phase for tandem mass spectrometry data acquisition. More than 40 fragments were identified, with excellent matches to calculated masses (Figure 5A,B; Table S2). From these, 22 IDE-cleavage sites of CCL4 were identified, clustered at several loop regions (Figure 5B). Those between aa 18-19, aa 22-23, aa 35-36, aa 43-44, and aa 62-63 occurred multiple times. When compared with cleavage sites of CCL4 and CCL3 by IDE, only two out of the five most frequent cleavage sites are shared despite their high sequence and structural similarity (Figure 5B, 5G).

To understand the molecular basis of CCL4 recognition by IDE, we solved the structure of CCL4 bound human IDE at 3.2Å (Table 1). In order to avoid cleavage of CCL4, E111Q was introduced to IDE to remove the catalytic base required for the protonation of a catalytic water. Crystals of IDE in complex with CCL4 were grown in conditions similar to those of IDE in complex with other substrates [7, 31-33], thus it had a similar space group, P6_5_, and unit cell dimensions (Table 1). The overall electron density for IDE is good despite the low resolution. The average B factor for IDE atoms is 52 Å^2^. The overall fold of the complex shows that, as in previously reported structures of substrate-bound IDE, IDE adopts a closed conformation (Figure 5C) [7, 31-33]. The electron density for CCL4 is relatively weak. We failed to detect CCL4 from the crystal by mass spectrometry, likely due to its small size. We modeled unfolded CCL4 into the existing density based on the presence of densities at the same locations in the previously reported substrate-bound IDE structures where we could successfully confirm the presence of substrates from the crystals by mass spectrometry [7, 31-33]. We only could model three N terminal residues of CCL4 at the exosite of IDE and 6 residues of CCL4 at the catalytic cleft of IDE. The average B factor of CCL4 is 83 Å^2^. We observed no electron density for the rest of CCL4.

At the exosite, the electron density corresponds to three N-terminal residues of CCL4, which form hydrogen bonds with the E341 carboxyl, and G361 and L359 carbonyl groups of IDE (Figure 5D). At the catalytic site, aa 42-47 of CCL4 are clearly visible (Figure 5E). We find that Q111 is in close proximity to the scissile bond of a major cleavage site, K45-R46, with the proper geometry for the proteolytic reaction. In comparison with CCL4 NMR structures [10], our structure of CCL4-bound IDE reveals that, in order to bind the catalytic chamber of IDE, both the N-terminus and the cleavage loop region of CCL4 must undergo a substantial conformational change upon their interaction with IDE (Figure 5F). Most noticeably, the α-helical region of residues 42-47 needs to transform into a β-strand to extend a β-sheet into the catalytic cleft of IDE (Figure 5E). When we mapped the major cleavage sites onto the CCL4 structure, we found four out of five sites that were in close proximity to one another (aa 18-19, aa 22-23, aa 43-44, and aa 62-63) (Figure 5B, 5G). The spatial clustering of the primary cleavage sites by IDE is also observed in CCL3 (Figure 5G).

The catalytic chamber of IDE is only large enough to engulf monomeric CCL4. Thus, we predict that IDE only degrades monomeric CCL4. As described above, a P8A mutation renders CCL4 predominantly monomeric without disturbing the integrity of its core structure (aa 10-69). We tested whether the P8A mutation makes CCL4 more readily degraded by IDE. MALDI-TOF mass spectrometry (MS) analyses demonstrated that IDE digests CCL4 P8A at a rate of ~9 s^−1^. This is significantly faster than wild type CCL4 (Figure S4) [7]. These data support the notion that IDE only degrades monomeric CCL4. Heparin has been shown to disrupt the polymerization of wild type CCL4, thus enhanced the degradation of CCL4 by IDE [7]. However, heparin did not noticeably enhance degradation of CCL4 P8A by IDE (not shown).

We have also shown that the degradation of CCL3 by IDE profoundly affects CCL3’s biological activities [7]. We thus investigated whether IDE could affect the chemotactic activity of CCL4 P8A in a human acute monocytic leukemia cell line, THP-1, using a modified Boyden chamber assay. We chose the CCL4 P8A mutant to control for the variable ability of IDE to degrade wild type CCL4, as it exists as a mixture of monomers, dimers, and higher order oligomers. We found that the treatment of CCL4 P8A with IDE shifted the concentration required for the peak of CCL4-mediated chemotaxis of THP-1 cells from 3 nM to 100 nM (Figure 5H). This indicates that cleavage of CCL4 by IDE leads to the inactivation of CCL4.

## Discussion

Our analyses reveal the structural basis of CC chemokine dimerization and oligomerization and highlight how subtle sequence differences in the N-terminal region of CCLs profoundly affect quaternary structures of CCLs. We find that the N-terminus of a CCL18 monomer within CCL18 dimer adopts α-helical conformation, which is distinct from the typical anti-parallel β-sheet shown in the other CCL dimers. Such an interface weakens the interaction between CCL18 monomers, explaining why CCL18 prefers the monomeric state in solution. Furthermore, the structure of CCL4 P8A reveals that the P8A mutation promotes the formation of an N-terminal type I β-turn, which prevents the formation of anti-parallel β-sheet essential for CCL4 dimerization. In the CCL3 structure, however, the P8A substitution disrupts crucial contacts between CCL3 dimers to reduce the formation of CCL3 polymers without affecting the dimer formation of CCL3.

Because P8A mutation can effectively prevent the dimerization of CCL2, CCL2 P8A mutant is a prototypical model to address the role of CCL oligomerization [6, 34-36]. CCL2 P8A can elicit various biological functions similar to wild type CCL2, e.g. receptor binding and inhibition of cAMP production in cultured cells [34] and CCL2-induced leukocyte recruitment [36]. However, CCL2 P8A is defective under certain experimental settings such as peritoneal cell recruitment [6]. In addition, CCL2 P8A has been shown to have anti-inflammatory properties [35]. Previous structural studies of CCL2 P8A mutant in complex with an antibody or herpesvirus decoy receptor have shown that the P8A mutation made the N-termini of CCL2 completely disordered [37, 38]. This is in part due to the abolishment of contacts between CCL2 Pro 8 and the hydrophobic groove of the other CCL2 monomer.

Together, these structures suggest that the P8A mutation could affect not only dimerization but also oligomerization and such effect is dependent upon the local sequence and contacts. As oligomerization of CC chemokines could affect the protease sensitivity, transcytosis and presentation, receptor-mediated leukocyte arrest, and chemokine-receptor interaction of CC chemokines, how the P8A mutation affects dimerization or oligomerization of CCLs may alter the biological outcome of these chemokines. Thus, precaution should be made in the use of P8A mutation to study the effect of dimerization and oligomerization of other CCLs.

In addition to the role of the N-terminus, the shape and surface charge of other regions of CCL also play vital roles in the oligomerization of CC chemokines. Despite their sequence similarity, CCL3/4, CCL5, and CCL18 form open-ended, rod shaped polymers in three distinct ways due to subtle changes on the contact surfaces (Figure S3). We also found that, for CCL18, formation of an open-ended polymer or tetramer is pH dependent. At physiological pH, both states may exist simultaneously, which would allow CCL18 to reversibly organize into a high-molecular weight complex (Figure 2C). In fact, we have observed the aggregation of CCL18 alone and with GAGs in solution via SAXS at pH 7.4. Such complexes may be functionally relevant as CCL18 is both constitutively and inducibly expressed to the high levels in various tissues normally and in disease conditions, such as cancer and inflammation [20, 24, 25]. Future studies are needed to address the molecular basis for the formation of these high molecular weight CCL18 aggregates and their functional relevance *in vivo*.

Our studies also elaborate the rules for the sensitivity of CCLs to degradation by IDE. We show that CCL18 is a poor substrate for IDE, even though CCL18’s preference for the monomeric state and its being, based on size alone, able to fit into the catalytic chamber of IDE. In conjunction with our previous report that CCL5 is also a poor substrate, we postulate that the high pI value of CCL18 leads to a charge mismatch between CCL18 and catalytic chamber of IDE at physiological pH. Since most CC chemokines have a high pI, it is likely that IDE negatively regulates monomeric CCL3 and CCL4 within the CC chemokine family [7]. As CCL3 and CCL4 are key proinflammatory chemokines and inflammation is key in the progression of human diseases, the link between IDE and chronic diseases such as Alzheimer’s disease may be in part due to the role of IDE in controlling the inflammation. Recently, several small molecules that can potently modulate the activity of IDE have been discovered and one shows promise in the treatment of diabetes [39-42]. Such reagents will allow the critical evaluation of whether and how IDE activity will impact CCL3 and CCL4-mediated immune responses during inflammation and infection and how such effects would impact IDE-based therapy.

## Experimental Procedures

### Cloning, expression and purification of recombinant proteins

A detailed protocol has been described previously for the expression and purification of CC chemokines [7], which has been modified as follows. The human CCL18 expression vector, pET32 was constructed with synthetic oligonucleotides, optimized for *E. coli* codon usage, and fused with a thioredoxin and a tobacco etch virus (TEV) cleavage site (ENLYFQS). A similar strategy was used for human CCL3 P8A, and CCL4 as described except an enterokinase cleavage site was used [7]. The expression vector for CCL4 P8A was generated by site-directed mutagenesis. All chemokines were expressed as hexahistidine-thioredoxin fusion proteins using *E. coli* BL21(DE3) and purified sequentially over Ni-NTA and source-Q columns. After proteolytic cleavage, the proteins were desalted and passed through a Ni-NTA column to remove the fusion partner and uncleaved chemokine. Intact chemokines were then purified to homogeneity over a heparin column. The catalytically inactive mutant of human cysteine free IDE, IDE-CF-E111Q was expressed and purified as described previously [7]. IDE-CF-E111Q in complex with CCL4 was generated by 5 cycles of co-incubation of two components followed by separation using an S200 size exclusion column. We have previously shown that repeated rounds of size exclusion chromatography is required to remove aggregated IDE and ensure the requisite purity of the IDE-substrate complex for crystallization [31].

### Protein crystallization and structure determination

Diffraction-quality crystals of CCL18, CCL4 P8A, CCL3 P8A, and IDE-CF-E111Q in complex with CCL4 were grown and optimized at 18°C by hanging drop vapor diffusion. Initial crystallization conditions were obtained from high-throughput crystallization screens with commercially available kits and the Mosquito® platform. Initial protein concentrations of 10 mg/ml CCL18, 6.2 mg/ml CCL3 P8A, 3.8 mg/ml CCL4 P8A, and 18-23 mg/ml IDE-CCL4 complex were mixed with the mother liquor at a 1:1 (v/v) ratio. The mother liquor for CCL18 contained 0.1 M sodium acetate trihydrate pH 4.6, and 2.0 M sodium chloride; that for CCL4 P8A was 15% ethanol (v/v), 0.1 M MES pH5.5, and 0.2 M Zn(OAc)_2_; that for CCL3 P8A was 1.26 M (NH_4_)_2_SO_4_,0.1M Na cacodylate pH 6.5; and that for IDE-CF-E111Q:CCL4 complex was 10-13% PEG MME 5000, 100 mM HEPES, pH 7.0, 10–14% Tacsimate, and 10% dioxane. CCL18 crystals were transferred into mother liquor containing 30% (w/v) sucrose and those of CCL3 P8A and CCL4 P8A were transferred into mother liquor containing 30% glycerol (v/v). Crystals of the IDE-CCL4 complex were first transferred to mother liquor with 15% glycerol and then with 30% glycerol. After cryoprotection, all crystals were flash frozen in liquid nitrogen.

X-ray diffraction data were measured at 100 K at beamline 19-ID at Advanced Photon Source (APS), Argonne National Laboratory (ANL) and processed with HKL3000. The structures of CCL18 and CCL3 P8A were solved by molecular replacement with PHASER [43] using the structure of CCL3 (PDB ID: 2X69) as the search model. Structures of CCL4 P8A and IDE-CF-E111Q were solved using CCL4 (PDB code: 2X6L) [7] and IDE (PDB code: 2G47) [31], respectively, as the search models. Structural refinement and rebuilding were performed with REFMAC [44], COOT [45], and PHENIX [46]. Electron densities corresponding to CCL4 at the exosite and catalytic chamber of IDE in the structures of IDE:CCL4 complex were clearly visible based on σ_A_-weighted Fo-Fc map calculated with REFMAC. CCL4 residues were manually assigned and built into the model with COOT. MolProbity [47] was used to validate model stereochemistry. Data collection and refinement statistics are summarized in Table 1. Chemokine interface analyses were performed with PISA [48].

### Small angle X-ray scattering (SAXS) Data Collection and Analysis

SAXS data for CCL18 in citrate buffer at pH 5.5 was collected at room temperature using a Mar 165 CCD detector at 18ID, APS, ANL using various protein concentrations (1-1.6 mg/ml) and an incident X-ray wavelength of 1.033Å. Data were reduced using custom macros for IgorPro (WaveMetrics, Inc.) written by the BioCAT staff and analyzed by ATSAS. SAXS data for CCL18 in bicine buffer at pH 9.0 was collected at room temperature at 12ID-B, APS, ANL using 1 mg/ml protein and an incident X-ray wavelength of 0.886 Å. The data was reduced and analyzed using ATSAS. PRIMUS [49] and GNOM [50] were used to determine the R_g_ value in reciprocal and real space, respectively. D_max_ and P(r) distribution were calculated by GNOM. Theoretical scattering curves for different models were generated and fit to the experimental data using CRYSOL [51]. OLIGOMER [52] was used to determine the percent composition by parsimonious conformational states that best fit the observed data. Data collection and scattering derived parameters are listed in Table S1 [53].

### Proteolytic cleavage of CC chemokines by IDE

Degradation of CCL4 and CCL4 P8A by IDE were performed at 37°C at a 50:1 chemokine/IDE molar ratio. After the indicated time, the reaction was stopped by the addition of an equal volume of stop buffer (200 mM EDTA and 0.2% trifluoroacetic acid). Reaction products were treated with 10 mM tris(2-carboxyethyl)phosphine (TCEP) for 15 min at room temperature and then desalted over a C18 ZipTip (Millipore) prior to MALDI-TOF mass spectrometry. For liquid chromatography-electrospray ionization-Fourier transform ion cyclotron resonance mass spectrometry in conjunction with the collision induced dissociation in the gas phase for tandem mass spectrometry data acquisition, enzyme reactions were carried out as described above in the presence of 0.8 mg/ml heparin. After the reactions were stopped and further treated with TCEP, samples (12μl) were injected into a nano RP-HPLC system (Dionex), with a C8 analytical column (Agilent). Peptides were eluted from the nano column with a linear gradient of 5-95% acetonitrile in 0.1% formic acid and sprayed into a LTQ-FT tandem MS instrument (Thermo Scientific). Spectra acquisition and analysis was performed as described [7]. MS and tandem MS/MS data were analyzed with web-based analysis tools FindPept, Mascot, and MassMatrix [54] and are summarized in Table S2.

### CCL4 chemotaxis assay

THP-1 human monocytic cells were maintained in RPMI1640 supplemented with 10% fetal bovine serum, 50 U/ml penicillin, 50μg/ml streptomycin, 50 μM β-mercaptoethanol, and 20 mM HEPES (Invitrogen). Migration of THP-1 cells in response to CCL4 P8A was measured in a 96-well MultiScreen-MIC filter plate (Millipore) with RPMI medium without phenol red and supplemented with 0.1% BSA. THP-1 cells (75μl 1.5 × 10^6^ cells/ml) were placed in the filter plate. CCL4 P8A proteins in the presence or absence of IDE treatment were placed in the lower chamber at the indicated concentrations. After a 4-hour incubation at 37°C, the upper 96-well filter plate was removed and the cells that had migrated to the lower receiver plate collected and quantified by a luminescence ATP detection assay system (PerkinElmer Life and Analytical Sciences, Waltham, MA) and counted on a Tecan Safire 2 microplate reader.

## Supplementary Material

1

2

3

4

5

6

## Figures and Tables

**Table 1:** Data collection and structure refinement statistics

	CCL18	CCL4-P8A	CCL3-P8A	IDE-CF-E111Q/CCL4
Data Collection				
Beamline	APS-19ID	APS 19ID	APS 19ID	APS 19ID
Wavelength (Å)	0.9792	0.9795	0.9795	0.9795
Space group	P2_1_	C2	P6_2_22	P6_5_
Cell dimension (Å)				
a	52.1	51.1	180.2	264.4
b	55.2	37.0	180.2	264.4
c	60.4	31.1	77.6	90.9
α	90.0	90	90	90
β	109.3	108.5	90	90
γ	90.0	90	120	120
Resolution (Å)	100 – 2.1	50-1.6	50-2.6	50-3.2
Rmeas (%)^a^	7.7 (63.9)^h^	11.3 (20.6)^h^	8.4 (54.2)^h^	23.8 (86.2)^h^
Rp.i.m (%)^b^	3.8 (31.4)^h^	6.0 (11.1)^h^	2.5 (16.2)^h^	11.2 (41.0)^h^
CC_1/2_^c^	(0.793)^h^	(0.955)^h^	(0.936)^h^	(0.768)^h^
CC*^d^	(0.941 )^h^	(0.988)^h^	(0.983)^h^	(0.932)^h^
I/sigma	19.4 (2.3)^h^	33.1 (9.6)^h^	40.5 (5.0)^h^	13.0 (3.1)^h^
Redundancy^e^	4.0 (3.8)^h^	5.0 (4.6)^h^	10.6 (11.1)^h^	3.2 (3.2)^h^
Completeness (%)	99.9 (99.7)^h^	95.6 (95.3)^h^	100.0 (100.0)^h^	95.5 (98.0)^h^
Unique reflections	19065	16016	21934	57680
Refinement				
R_work_^f^	0.176	0.166	0.204	0.192
R_free_^g^	0.218	0.213	0.248	0.255
No. atoms				
Protein	2087	1083	2637	15756
Water	195	167	54	67
B-factors				
Protein	29.3	23.4	54.2	51.7
Substrate	-	-	-	82.6
Water	37.4	27.8	43.3	39.2
r.m.s. deviations				
Bond lengths (Å)	0.007	0.007	0.004	0.004
Bond angles (°)	1.061	1.092	0.744	0.848
Ramachandran plot (%)				
Favorable region	96.4	93.3	94.8	90.8
Allowed region	3.6	6.7	5.2	9.2
Generously allowed region	0	0	0	0
Disallowed region	0	0	0	0
PDB code	4MHE	3TN2	4RA8	4RAL

a *R*_meas_ = Σ_hkl_ [n/(n-1)]^1/2^Σ_i_| I_hkl,I_ - <I_hkl_>|/Σ_hkl_ <I_hkl_>

b *R*_p.i.m._ = Σ_hkl_ [1/(n-1)]^1/2^ Σ_i_|I_hkl,I_ - <I_hkl_>|/Σ_hkl_ <I_hkl_>

c CC_1/2_ – Pearson correlation coefficient between random half-datasets - *ρ*_x,y_=cov*[(x,y)/(σ_x_σ_y_)]*

d CC*=[2CC_1/2_/(1+CC_1/2_)]^1/2^

e *N*_obs_/*N*_unique_

f *R*_work_ = Σ_hkl_ ||F_obs_| - *k* |F_calc_||/ Σ_*hkl*_ |F_obs_|

g *R*_free_, calculated the same as for **R**_work_ except 5% data were excluded from the refinement calculation.

h the outer resolution shell. Values in parentheses indicate the highest resolution shell

## Highlights

- Human CCL18 structure and SAXS study reveal why CCL18 prefers the monomeric state.
- Position 8 determines CC chemokine oligomerization.
- Quaternary and tertiary structures dictate the susceptibility of CCL3, CCL4, and CCL18 to insulin degrading enzyme.

## Abbreviations

IDE: insulin degrading enzyme
MIP: macrophage inflammatory protein
CCL: CC chemokine ligand
SAXS: small angle X-ray scattering
PDB: protein data bank
GAG: glucosaminoglycan
APS: Advanced Photon Source
ANL: Argonne National Laboratory
MW: molecular weight
