# Supplementary material for: Structures of human CCL18, CCL3, and CCL4 reveal molecular determinants for quaternary structures and sensitivity to insulin degrading enzyme
Source: J Mol Biol. Author manuscript; Available in PMC 2016 Mar 27. (PMC4355285; doi:10.1016/j.jmb.2015.01.012)
Supplement: 6 [file NIHMS661239-supplement-6.pdf]

Supplemental figure legends

Figure S1 SAXS data of CCL18. (A) Scattering curve (left) and Guinier plot (right) of CCL18 at pH=7.4 in phosphate buffer saline. The curve-up feature in the plot of ln[I(q)] vs q^2 (instead of linear one as in B) shows the formation of severe non-specific aggregation under this buffer condition. (B) Guinier plots of SAXS data of CCL18 in 20 mM citrate buffer, pH=5.5 (left) and in 20 mM bicine buffer, pH=9.0 (right).

Figure S2 Detailed SAXS analyses that exclude various probable models. For SAXS data using citrate buffer (pH=5.5), we compared the best fit (model using the mixture of monomer, dimer, and tetramer (M1+D1+T1)) to the experimental data with those fits resulting from use of (A) monomer alone (M1), dimer alone (D1), or tetramer alone (T1) and (B) those using the mixture of monomer (M1), dimer (D1), and three probable tetramer states (T1-T3 from CCL18, T4 from CCL3 and T5 from CCL5) (B). For SAXS data using bicine buffer (pH=9), we compared the best fit (model using the mixture of monomer, dimer, and hexamer (M1+D2+H2)) with those fits resulting from using (C) monomer alone (M1), dimer alone (D2), or hexamer alone (H2) and with those from using (D) the mixture of monomer (M1) and dimer (D2) in addition to two different probable tetramers (T1, T2), or four different hexamers (H1-H2 from CCL18, H3 from CCL3, and H4 from CCL5). The structures of different CCL18 states are shown by ribbon representation at the bottom. Models used to fit the SAXS data were generated from structures of CCL18 (4MHE), CCL3 (2X69) and CCL5 (2L9H).

Figure S3 Comparison of CCL18, CCL5, and CCL3/CCL4 polymers. Each dimer of CCL3/4, CCL5 and CCL18 is depicted by ribbon representation (top and middle) and surface charge (bottom). PDB codes for CCL3, CCL5, and CCL18 are 2X69, 2L9H, 4MHE, respectively. Electrostatic surfaces are calculated with APBS [51] and depicted in red for negatively charged surface and blue for positively charged ones (-1kT to 1kT).

Figure S4 MALDI-MS profiles of four peptides, insulin, CCL18, CCL4, and CCL4 P8A in the presence or absence of IDE. Peptide degradation by IDE was performed at 37°C with a 50:1 chemokine/IDE molar ratio for CCL4 and 200:1 ratio for CCL18 for 5 seconds.

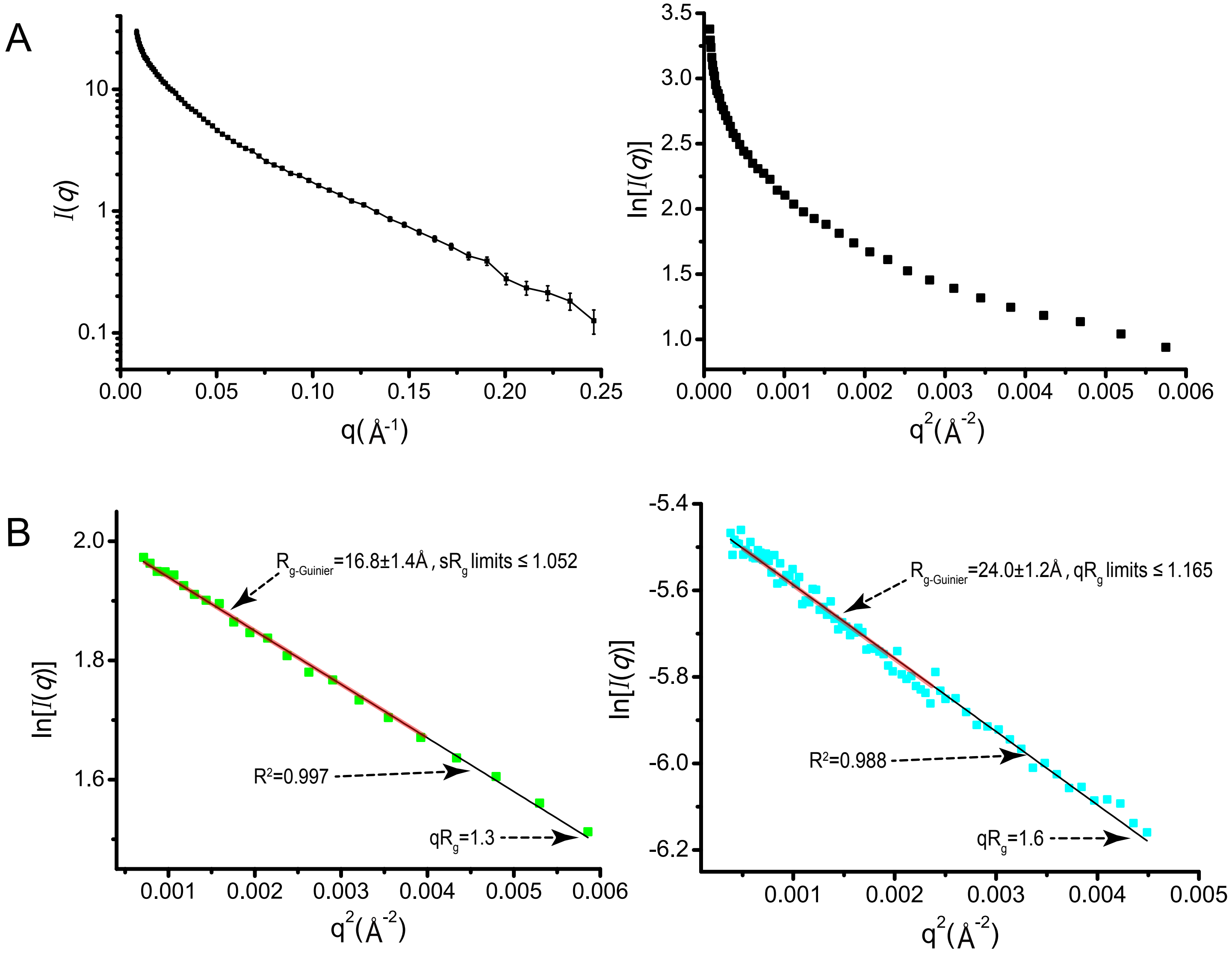
A
B
I(q)
q(Å⁻¹)
ln[I(q)]
q²(Å⁻²)
R g-Guinier=16.8±1.4Å , sRg limits ≤ 1.052
R²=0.997
qRg=1.3
R g-Guinier=24.0±1.2Å , qRg limits ≤ 1.165
R²=0.988
qRg=1.6

Figure S2

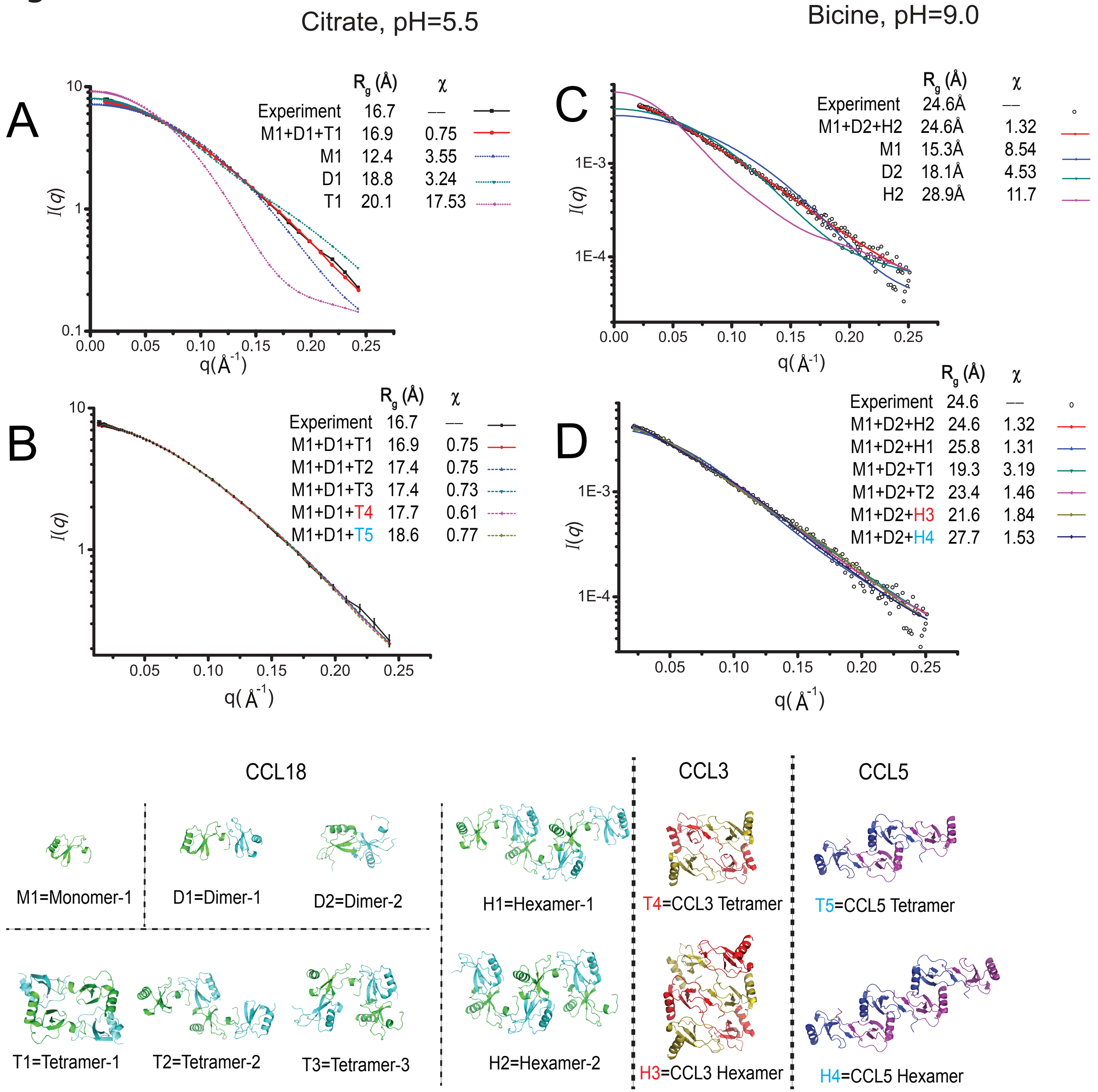

Citrate, pH=5.5
Bicine, pH=9.0
A
Rg (Å) χ
Experiment 16.7 —
M1+D1+T1 16.9 0.75
M1 12.4 3.55
D1 18.8 3.24
T1 20.1 17.53
I(q)
q(Å-1)
B
Rg (Å) χ
Experiment 16.7 —
M1+D1+T1 16.9 0.75
M1+D1+T2 17.4 0.75
M1+D1+T3 17.4 0.73
M1+D1+T4 17.7 0.61
M1+D1+T5 18.6 0.77
C
Rg (Å) χ
Experiment 24.6Å —
M1+D2+H2 24.6Å 1.32
M1 15.3Å 8.54
D2 18.1Å 4.53
H2 28.9Å 11.7
D
Rg (Å) χ
Experiment 24.6 —
M1+D2+H2 24.6 1.32
M1+D2+H1 25.8 1.31
M1+D2+T1 19.3 3.19
M1+D2+T2 23.4 1.46
M1+D2+H3 21.6 1.84
M1+D2+H4 27.7 1.53
CCL18
CCL3
CCL5
M1=Monomer-1
D1=Dimer-1
D2=Dimer-2
H1=Hexamer-1
T4=CCL3 Tetramer
T5=CCL5 Tetramer
T1=Tetramer-1
T2=Tetramer-2
T3=Tetramer-3
H2=Hexamer-2
H3=CCL3 Hexamer
H4=CCL5 Hexamer

Figure S3

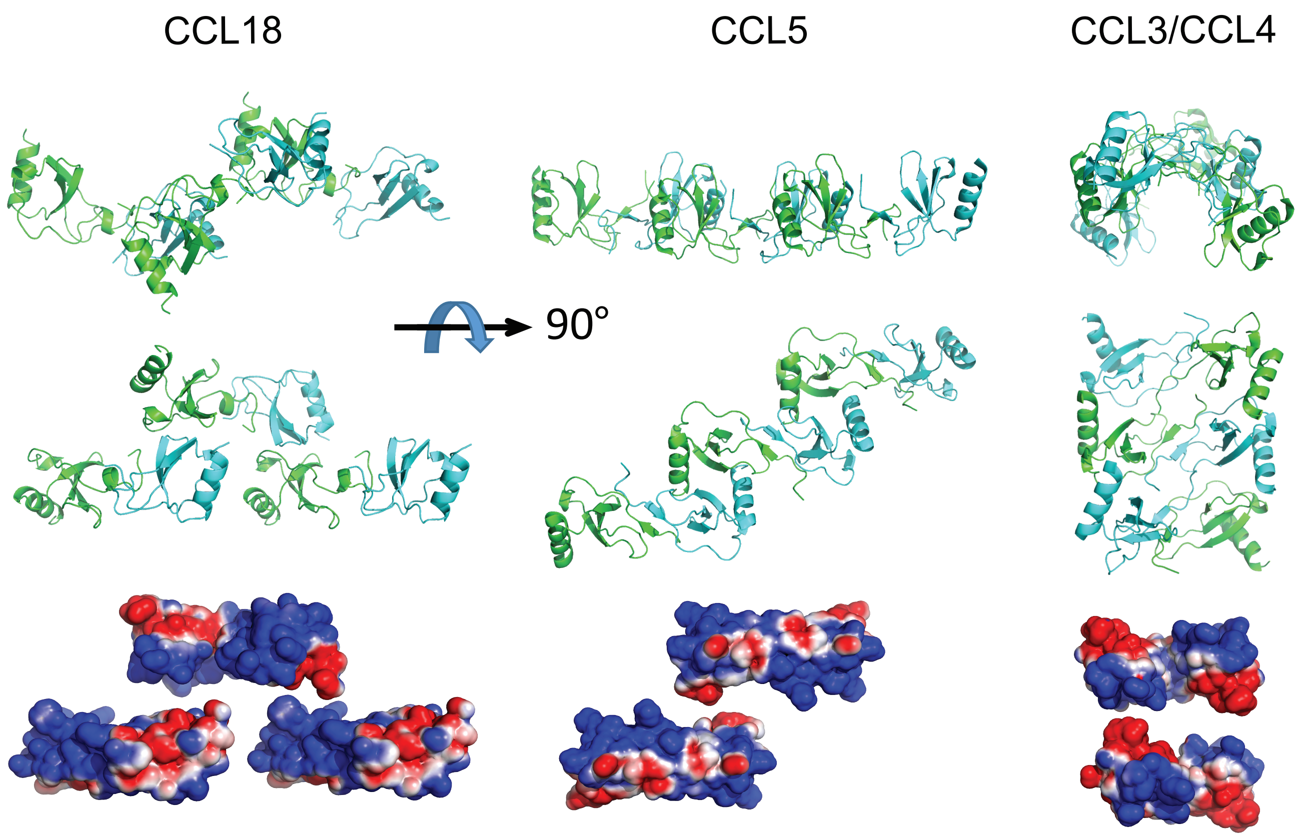

Figure S4

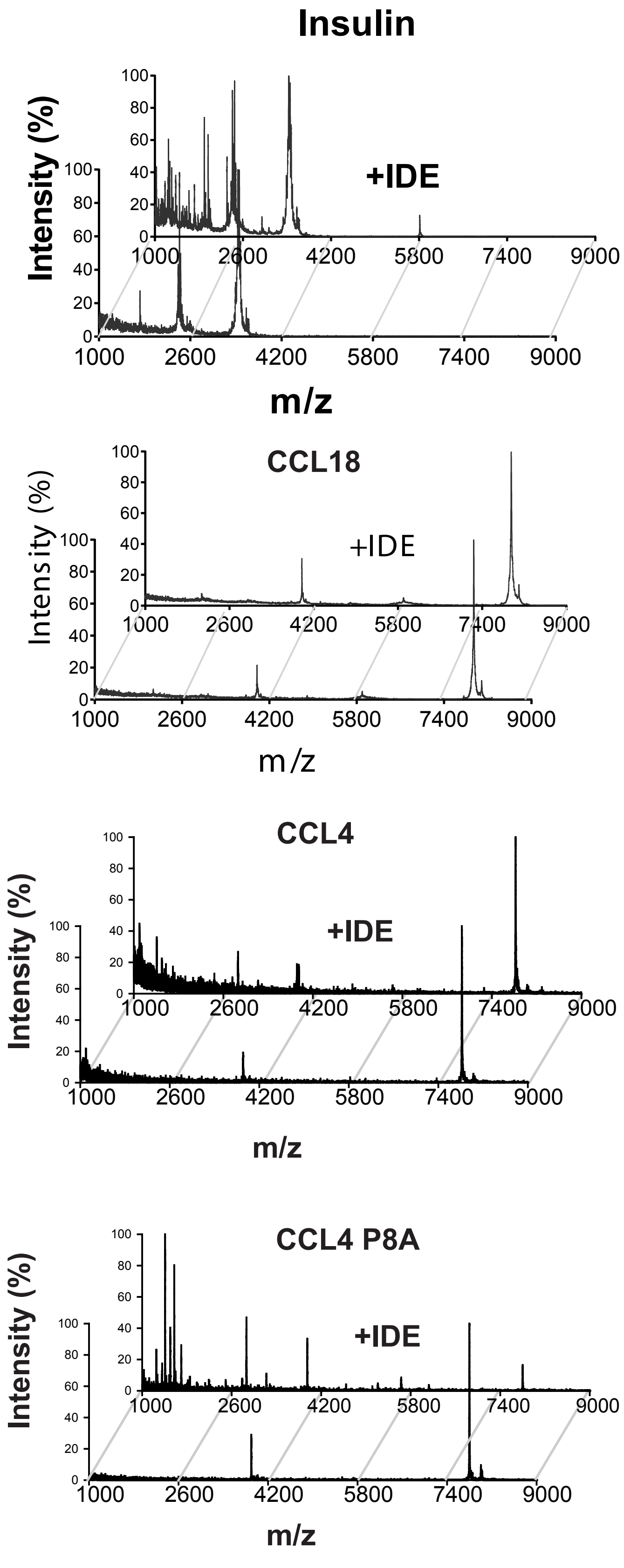
Insulin
+IDE
Intensity (%)
100
80
60
40
20
0
1000
2600
4200
5800
7400
9000
m/z
CCL18
+IDE
Intensity (%)
m/z
CCL4
+IDE
Intensity (%)
m/z
CCL4 P8A
+IDE
Intensity (%)
m/z

Table S1 SAXS data collection and scattering-derived parameters

	CCL18 in citrate	CCL18 in PBS	CCL18 in bicine
**Data-collection parameters**			
Instrument	18ID (BioCAT)	18ID (BioCAT)	12ID-B (APS)
Wavelength (Å)	1.033	1.033	0.886
q range (Å -1)	0.013-0.243	0.008-0.246	0.021-0.251
Exposure time (s)	1	1	1
Number of frames collected	16	20	30
Concentrations (mg/ml)	1, 1.5	1	1
Temperature (℃)	24	24	23
**Structural parameters**			
I(0) [from P(r)]	7.6 ± 0.1		(4.5±0.1)E-03
Rg (Å) [from P(r)]	16.7 ± 0.1		24.6±0.1
I(0) (from Guinier)	7.7 ±0.1		0.005±0.000
Rg (Å) (from Guinier)	16.8 ± 1.4		24.0±1.2
Dmax[a] (Å)	58±3		90±7
**Software employed**			
Primary data reduction	Igor Pro	Igor Pro	Matlab & Igor Pro
Data processing	ATSAS	ATSAS	ATSAS
Oligmeric component fitting	OLIGOMER		OLIGOMER
Three-dimensional graphics representations	PyMOL		PyMOL

[a]Dmax is a model parameter in the P(r) calculation, the error is an estimate based on the results of P(r) calculations using a range of Dmax values.

Table S2: Summary of mass spectrometry data of IDE-digested CCL4 by FT-ICR.

Peptide	M_{Cal} (M)	M_{Obs} (M)	|Δppm|	MS/MS (b/y ions)
1-69	7809.630	7809.681	6.50	
1-12	1149.437	1149.434	2.61	b5-6,8-11; b^{++}10-11; y2,6,7; y^{++}10-11
1-13	1296.505	1296.504	0.77	b5-6,9-12; b^{++}11-12; y2-3,6-8
1-14	1383.538	1383.534	2.89	b5-6,10-13; b^{++}12-13; y2-6,8-9,11; y^{++}11-13
1-18	1874.787	1874.792	2.67	b3-17; y3-16
1-19	2002.882	2002.876	3.00	b3, 6-7,9-10,12,18; b^{++}13,18; y4-6,8-10,13; y^{++}4,6,8,12-18;
1-21	2213.018	2213.013	2.26	b3-12; , b^{++}11-14,16,18-20; y3,5-8,14; y^{++}7-20
1-22	2369.120	2369.123	1.27	b3-7,9-12; b^{++}8-12,14,16; y2,4,6-7; y^{++}7-16
1-35	3889.769	3889.818	12.6	b6,10,12; b^{++}18,20,23; y8-10; y^{++}15-16,18-21
13-21	1082.599	1082.596	2.77	b2-8; b^{++}6-8; y2-7; y^{++}6-8
13-24	1499.812	1499.809	2.00	b2-8; b^{++}6-11; y3-5; y^{++}4-11
13-27	1812.976	1812.974	1.10	b2,3,5-7; b^{++}3,6-9,11-14; y2,4-8,14; y^{++}7-14
13-35	2759.350	2759.352	0.72	b6,10,12; b^{++}18,20,23; y8-10; y^{++}15-16,18-21
17-27	1314.764	1314.775	8.37	b2; b^{++}6-10; y1-2,4-5
18-28	1406.790	1406.786	2.84	b2-4,6-7; b^{++}4-10; y1-5,7-8; y^{++}3-4,6-9
19-27	1087.626	1087.623	2.76	b2-8; b^{++}6-8; y2-8; y^{++}7-8
19-35	2034.000	2034.002	0.98	b5-12; b^{++}12-16; y6-8,15-16; y^{++}11-12,14-16
19-45	3119.587	3119.619	10.26	b7-12; b^{++}9-26; y3-6,8-15,17; y^{++}15-18,25
19-46	3275.688	3275.697	2.75	b4-11; b^{++}8-24; y4-13; y^{++}9-21,25
19-48	3490.815	3490.817	0.57	b4,6,7; b^{++}8-17; y5,18; y^{++}10-18
22-28	912.457	912.455	2.19	b2-6; b^{++}2,4-6; y1-5; y^{++}6
22-33	1479.676	1479.675	0.68	b4-10; b^{++}2,4-6; y1-5; y^{++}6
22-34	1592.759	1592.759	0.38	b3-12; b^{++}8-12; y3-11; y^{++}8,10,12
22-35	1695.768	1695.776	4.72	b2-12; b^{++}9-13; y3-12; y^{++}11-13
22-46	2937.456	2937.458	0.68	b4-8,16-17; b^{++}7-16,18; y4-9; y^{++}6,9-12,14-19,24
23-45	2625.254	2625.265	4.19	b6-7,9-15,17-18; b^{++}13,15,21; y4-6,8-11,13-17; y^{++}18,20
23-46	2781.355	2781.368	4.67	b2-13,15,18; b^{++}6-8,15-16,18-19; y5-11,14; y^{++}6-7,9-22
23-48	2996.482	2996.490	2.67	b3-9,12-13,18; b^{++}6,9,14,16-17,19,25; y6-11,14,18; y^{++}8-9,11-24
28-42	1693.778	1693.780	1.18	b4,7-8,10,12-14; b^{++}13-14; y2,5-7,8-9,11; y^{++}14
28-45	2050.979	2050.979	0.15	b5-10,12-17; b^{++}11,16-17; y3-15,17; y^{++}6,8,11,15-17
29-42	1530.714	1530.716	1.31	b4-9,11-13; b^{++}8,12-13; y2,4-9,11; y^{++}5,9,13
29-45	1887.916	1887.921	2.65	b3,5-16; b^{++}6,11,14,16; y3-16; y^{++}7,9,11,14,15
36-42	747.403	747.400	4.01	b3-6; y3-5
36-45	1104.605	1104.601	3.62	b2-9; b^{++}7-8; y2-8; y^{++}8-9
36-46	1260.706	1260.703	2.38	b2,4-10; y4-9; y^{++}6-10
36-48	1475.833	1475.831	1.36	b6,7,11; b^{++}11-12; y4-8,11; y^{++}9-11
36-57	2392.193	2392.189	1.67	b3,6,11,17,18; b^{++}8,13-21; y3-5,7-10,17-18; y^{++}12-21
36-69	3943.917	3943.938	5.32	b7,9,11; b^{++}13,15-17,19-24; y5-13,16; y^{++}11-12,14-15,16-19,21
43-61	2206.055	2206.048	3.17	b8; b^{++}4,9,11-18; y2-5,7-8,13,16-17 y^{++}12,14,16,18
43-69	3215.531	3215.535	1.24	b4,6,8,17; b^{++}8-9,11-18; y2,4-13,16; y^{++}8-13,15-17
46-61	1848.855	1848.846	4.87	b3-14; b^{++}11-15; y3-5,7-14; y^{++}8-10,13,15
46-69	2858.330	2858.345	5.25	b5-8,12,17; b^{++}8,10-23; y3-13,16; y^{++}6,10,13,21,22
47-67	1692.753	1692.748	2.95	b3-4,6-13; b^{++}13,14; y2-4,8-9,11; y^{++}11
47-69	2702.229	2702.260	11.47	b4-7,9-17; b^{++}17-22; y4-16; y^{++}10,14,17,20
49-61	1477.626	1477.623	2.03	b4-12; b^{++}5,10-12; y2-6,8-10; y^{++}11-12
58-69	1570.742	1570.750	5.09	b2,4-11; b^{++}10-11; y2-10; y^{++}6,10-11
